# Perceptions of Harm to Children Exposed to Secondhand Aerosol From Electronic Vapor Products, Styles Survey, 2015

**DOI:** 10.5888/pcd14.160567

**Published:** 2017-05-25

**Authors:** Kimberly H. Nguyen, Van T. Tong, Kristy Marynak, Brian A. King

**Affiliations:** 1 Office on Smoking and Health, National Center for Chronic Disease Prevention and Health Promotion, Centers for Disease Control and Prevention, Atlanta, Georgia; 2 Division of Reproductive Health, National Center for Chronic Disease Prevention and Health Promotion, Centers for Disease Control and Prevention, Atlanta, Georgia

## Abstract

**Introduction:**

The US Surgeon General has concluded that e-cigarette aerosol is not harmless and can contain harmful and potentially harmful chemicals, including nicotine. We assessed factors associated with adults’ perceptions of harm related to children’s exposure to secondhand aerosol from electronic vapor products (EVPs).

**Methods:**

Data came from the 2015 Styles, an Internet panel survey of US adults aged 18 years or older (n = 4,127). Respondents were asked whether they believe aerosol from other people’s EVPs causes children harm. Harm perceptions were assessed overall and by cigarette smoking, EVP use, and sociodemographic characteristics. Multinomial logistic regression was used to assess odds of perceived harm.

**Results:**

Overall, 5.3% of adults responded that secondhand EVP exposure caused “no harm” to children, 39.9% responded “little harm” or “some harm,” 21.5% responded “a lot of harm,” and 33.3% responded “don’t know.” Odds of “no harm” response were greater among men than among women, current and former cigarette smokers than among never smokers, and current and former EVP users than among never users; odds were lower among non-Hispanic blacks, Hispanics, and non-Hispanic other races than among non-Hispanic whites. Odds of responding “don’t know” were greater among men, current cigarette smokers, and current and former EVP users; odds were lower among those aged 45 to 64 years than those aged 18 to 24 years and lower among non-Hispanic other races and Hispanics than non-Hispanic whites.

**Conclusion:**

Two-fifths of US adults believe that children’s exposure to secondhand EVP aerosol causes some or little harm, while one-third do not know whether it causes harm. Efforts are warranted to educate the public about the health risks of secondhand EVP aerosol, particularly for children.

**MEDSCAPE CME**

Medscape, LLC is pleased to provide online continuing medical education (CME) for this journal article, allowing clinicians the opportunity to earn CME credit.

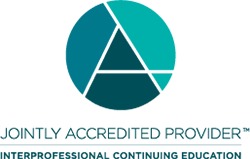
In support of improving patient care, this activity has been planned and implemented by Medscape, LLC and Preventing Chronic Disease. Medscape, LLC is jointly accredited by the Accreditation Council for Continuing Medical Education (ACCME), the Accreditation Council for Pharmacy Education (ACPE), and the American Nurses Credentialing Center (ANCC), to provide continuing education for the healthcare team.

Medscape, LLC designates this Journal-based CME activity for a maximum of 1.00 *AMA PRA Category 1 Credit(s)™*. Physicians should claim only the credit commensurate with the extent of their participation in the activity.

All other clinicians completing this activity will be issued a certificate of participation. To participate in this journal CME activity: (1) review the learning objectives and author disclosures; (2) study the education content; (3) take the post-test with a 75% minimum passing score and complete the evaluation at http://www.medscape.org/journal/pcd; (4) view/print certificate.

**Release date: May 25, 2017; Expiration date: May 25, 2018**

**Learning Objectives**

Upon completion of this activity, participants will be able to:

1. Distinguish the percentage of adults who believe that secondary aerosols from electronic vapor products (EVPs) represent little or no health risk
2. Distinguish the percentage of adults who believe that secondary aerosols from EVPs represent a significant health risk
3. Evaluate variables associated with attitudes toward secondary aerosols from EVPs
4. Analyze the potential harm associated with secondary aerosols from EVPs

**EDITOR**

Caran Wilbanks

Editor, Preventing Chronic Disease

Disclosure: Caran Wilbanks has disclosed the following relevant financial relationships:

Other: Partner is employed by McKesson Corporation

**CME AUTHOR**

Charles P. Vega, MD

Health Sciences Clinical Professor, UC Irvine Department of Family Medicine; Associate Dean for Diversity and Inclusion, UC Irvine School of Medicine, Irvine, California

Disclosure: Charles P. Vega, MD, has disclosed the following relevant financial relationships:

Served as an advisor or consultant for: McNeil Consumer Healthcare

Served as a speaker or a member of a speakers bureau for: Shire Pharmaceuticals

**AUTHORS**

Kimberly H. Nguyen, MS, MPH

Office on Smoking and Health, National Center for Chronic Disease Prevention and Health Promotion, Centers for Disease Control and Prevention, Atlanta, Georgia, USA

Disclosure: Kimberly H. Nguyen, MS, MPH, has disclosed no relevant financial relationships.

Van Tong, MPH

Division of Reproductive Health, National Center for Chronic Disease Prevention and Health Promotion, Centers for Disease Control and Prevention, Atlanta, Georgia, USA

Disclosure: Van Tong, MPH, has disclosed no relevant financial relationships.

Kristy Marynak, MPH

Office on Smoking and Health, National Center for Chronic Disease Prevention and Health Promotion, Centers for Disease Control and Prevention, Atlanta, Georgia, USA

Disclosure: Kristy Marynak, MPH, has disclosed no relevant financial relationships.

Brian A. King, PhD, MPH

Office on Smoking and Health, National Center for Chronic Disease Prevention and Health Promotion, Centers for Disease Control and Prevention, Atlanta, Georgia, USA

Disclosure: Brian A. King, PhD, MPH, has disclosed no relevant financial relationships.

## Introduction

Electronic vapor products (EVPs), also called electronic nicotine delivery systems, are devices designed to deliver nicotine and other additives to the user in the form of an aerosol, which is then exhaled by the user into the environment. Depending on the brand, EVP cartridges typically contain nicotine, a component to produce the aerosol (eg, propylene glycol or glycerol), and flavorings (eg, fruit, mint, chocolate). There are multiple types of EVPs on the US market, including e-cigarettes, e-hookahs, hookah pens, vape pens, and e-cigars; some of these products are disposable, while others can be refilled or recharged for repeated use. Some EVP marketing claims that they emit “harmless water vapor” (1,2) However, scientific evidence indicates that the aerosol emitted by EVPs may expose nonusers, including children and infants, to aerosolized nicotine and other potentially harmful substances, including heavy metals, ultrafine particulates, and volatile organic compounds; thus, this aerosol is not as safe as clean air (3). The US Surgeon General has concluded that e-cigarette aerosol is not harmless and that it can contain harmful and potentially harmful chemicals, including nicotine (4). Animal and human studies found that fetal and adolescent nicotine exposure can result in adverse health effects, including impaired brain and lung development (4,5). Nicotine exposure during pregnancy could result in low birthweight, preterm delivery, stillbirth, and sudden infant death syndrome (4).

Children and infants can be exposed to nicotine from secondhand EVP aerosol in numerous ways. Components of exhaled EVP aerosol are released into the atmosphere where it can be inhaled by others (2). These components can also settle on surfaces, where they can be ingested, inhaled, or absorbed through the skin by children and infants (2,3). Children and infants can be exposed to secondhand aerosol in numerous places, including private settings such as homes and vehicles as well as public places such as restaurants (5). Infants and children may be especially vulnerable to the possible harmful effects of substances in EVP aerosol in lower concentrations because of their smaller body weight and developing respiratory systems (3,5). Nicotine is toxic to infants and children if ingested, inhaled, or absorbed through skin. Additionally, nicotine exposure during periods of developmental vulnerability, from fetal through adolescent stages, can have multiple adverse health effects, including impaired brain and lung development (6).

Some studies indicate that young people and adults perceive EVPs as a safer alternative to conventional cigarettes, and some e-cigarette users perceive these products as a substitute for conventional cigarettes in places where smoking is not allowed (7–9). A 2014 analysis found that 88% of e-cigarette websites noted that they can be used anywhere, with 71% talking about the circumvention of public smoke-free laws (1). The potential for involuntary secondhand exposure to EVP aerosol among nonusers, children and adults alike, in public indoor environments is of increasing concern given that use of EVPs has increased considerably among US youths and adults in recent years (10,11).

One study of adults’ perceptions of harm from secondhand EVP aerosol was conducted in Spain during 2013 through 2014; it found that nearly 70% of those surveyed believed that aerosol from EVP exposure was not harmful to nonusers in general (12). However, to our knowledge, no national study has examined perceived harm from secondhand EVP exposure in the United States among either adults or children. Because of the higher potential vulnerability of children to secondhand EVP exposure and the importance of determining population groups that will benefit most from public health messaging and interventions to reduce secondhand EVP exposure, we assessed perceptions of harm related to children’s exposure to EVP aerosol among a nationally representative sample of US adults.

## Methods

### Data source

Data came from 2015 Styles, a series of seasonal national consumer surveys developed and fielded by Porter Novelli. Styles draws from KnowledgePanel, an Internet panel recruited by using address-based probability sampling to reach respondents regardless of landline telephone or Internet access (10). If needed, households are provided with a laptop computer and Internet access. Styles is conducted among a nationally representative sample of US adults aged 18 years or older. In 2015, a total of 4,127 participants completed Styles, a 67% response rate. Data were weighted to the US Census Current Population Survey distributions by using 9 factors: sex, age, annual household income, race/ethnicity, household size, education level, US Census region, whether the respondent resided in a metropolitan statistical area, and whether the respondent had Internet access before the start of the survey (13). Data were de-identified for analysis, and the study was determined by the Centers for Disease Control and Prevention’s Institutional Review Board to be exempt from human subjects review.

### Measures

Adults’ harm perceptions related to children’s exposure to secondhand aerosol were assessed by the question “Do you believe that when children breathe the aerosol from other people’s electronic vapor products that it causes . . . ?” Reponses options were “no harm,” “little harm,” “some harm,” “a lot of harm,” and “don’t know.” This measure was developed using established measures of tobacco-related harm perceptions, in consultation with subject matter experts, and subsequently cognitively tested to ensure validity.

Current cigarette smokers were respondents who reported smoking at least 100 cigarettes during their lifetime and who smoked cigarettes every day or some days at the time of the survey. Former smokers were respondents who reported having smoked at least 100 cigarettes in their lifetime and who smoked not at all at the time of survey. Never cigarette smokers were respondents who reported not having smoked 100 cigarettes in their lifetime.

Current EVP users were respondents who reported using any of the following EVPs in their lifetime and within the previous 30 days: “electronic cigarettes (e-cigarettes), such as Blu, 21st Century Smoke, or NJOY”; “electronic hookahs (e-hookahs), hookah pens, or vape pens, such as Starbuzz or Fantasia”; or “some other electronic vapor product such as electronic cigars (e-cigars) or electronic pipes (e-pipes).” Former EVP users were respondents who reported using EVPs at least once in their lifetime and who reported not using EVPs in the previous 30 days. Never EVP users were respondents who reported not using EVPs in their lifetime and not using EVPs in the previous 30 days. Four (<1%) respondents were excluded from the analyses because they answered that they had never used EVPs in their lifetime but had used EVPs in the previous 30 days.

Assessed sociodemographic variables were sex, age, race/ethnicity, educational attainment, annual household income, marital status, US Census region, and whether at least 1 child younger than 18 years lived in the respondent’s household.

### Analysis

Descriptive statistics were calculated to assess the distribution of harm perceptions by cigarette smoking, EVP use, and sociodemographic characteristics. We used χ^2^ tests to assess significant differences between groups (*P* < .05). Adjusted multinomial logistic regression was conducted to determine the odds of “no harm,” “little harm” or “some harm,” and “don’t know” responses compared with “a lot of harm” response. The adjusted model included all of the aforementioned cigarette smoking, EVP use, and sociodemographic characteristics. Analyses were conducted by using SAS version 9.3 (SAS Institute, Inc), and data were weighted to adjust for selection and nonresponse.

## Results

A total of 4,127 adults completed the survey. The sociodemographic distribution of respondents was as follows: women, 52%; aged 45 to 64 years, 35%; non-Hispanic white, 66%; high school graduates, 30%; annual household income of $50,000 to $99,999, 35%; married, 60%; live in the South, 37%; and no children younger than 18 years living in the household, 72%. Approximately 14% were current cigarette smokers and 5% were current EVP users.

Overall, 5.3% responded that children’s exposure to secondhand aerosol from EVPs caused “no harm,” 39.9% responded “little harm” or “some harm,” 21.5% responded “a lot of harm,” and 33.3% responded “don’t know” (Table 1). Significant variation in harm perceptions were observed by sex, age, race/ethnicity, educational attainment, annual household income, US Census region, cigarette smoking status, and EVP use (*P* < .05).

**Table 1:** Perceptions of Harm for Children Exposed to Secondhand Aerosol from Electronic Vapor Products (EVPs), US Adults Aged 18 Years or Older, by Sociodemographic Characteristics, Cigarette Smoking Status, and EVP Use, Styles Survey,^a^ 2015

Characteristic	Total, n (%)^c^	Perceived Harm^b^			
		No Harm, % (95% CI)	Little Harm or Some Harm, % (95% CI)	A Lot of Harm, % (95% CI)	Don’t Know, % (95% CI)
**Overall**	4,127 (100.0)	5.3 (4.5–6.0)	39.9 (38.2–41.7)	21.5 (20.0–23.1)	33.3 (31.6–35.0)
**Sex**					
Female	2,218 (51.8)	3.7 (2.8–4.6)^d^	37.6 (35.2–40.0)	25.2 (23.0–27.3)	33.5 (31.2–35.8)
Male	1,909 (48.2)	6.9 (5.6–8.2)	42.4 (39.8–45.0)	17.7 (15.6–19.8)	33.0 (30.6–35.5)
**Age, y**					
18–24	299 (12.5)	5.1 (2.4–7.9)^d^	48.4 (42.2–54.5)	21.0 (15.9–26.1)	25.5 (20.3–30.8)
25–44	1,171 (34.1)	5.1 (3.6–6.5)	43.5 (40.2–46.8)	21.3 (18.5–24.1)	30.1 (27.1–33.2)
45–64	1,799 (34.7)	6.1 (4.8–7.3)	38.4 (35.8–41.0)	19.9 (17.6–22.1)	35.7 (33.1–38.3)
≥65	585 (18.7)	4.2 (2.7–5.7)	30.6 (27.2–33.9)	25.5 (22.2–28.8)	39.8 (36.1–43.4)
**Race/ethnicity**					
Non-Hispanic white	3,083 (65.6)	6.1 (5.2–7.1)^d^	40.1 (38.1–42.1)	17.7 (16.2–19.3)	36.0 (34.1–38.0)
Non-Hispanic black	399 (11.6)	4.2 (2.0–6.3)	37.2 (31.8–42.6)	24.7 (19.7–29.6)	34.0 (28.7–39.3)
Hispanic	444 (15.1)	3.9 (1.6–6.3)	38.8 (33.6–44.1)	29.6 (24.7–34.4)	27.7 (22.9–32.4)
Non-Hispanic other than white or black	201 (7.7)	^—^ e	44.2 (36.2–52.3)	33.7 (25.9–41.6)	20.1 (13.7–26.5)
**Education level**					
<High school graduate	292 (12.2)	8.2 (4.7–11.6)^d^	33.2 (27.1–39.4)	22.9 (17.6–28.3)	35.6 (29.5–41.8)
High school graduate	1,232 (29.7)	5.7 (4.3–7.2)	36.6 (33.5–39.7)	20.9 (18.2–23.6)	36.8 (33.7–39.8)
Some college	1,257 (28.8)	5.4 (4.0–6.8)	42.7 (39.4–45.9)	20.3 (17.6–23.0)	31.7 (28.7–34.7)
College degree or beyond	1,346 (29.3)	3.4 (2.4–4.5)	43.3 (40.2–46.4)	22.9 (20.2–25.6)	30.4 (27.5–33.2)
**Annual household income, $**					
<25,000	739 (17.9)	7.6 (5.2–10.0)^d^	34.2 (30.1–38.2)	19.5 (16.0–22.9)	38.8 (34.6–43.0)
25,000–49,999	1,082 (22.4)	4.7 (3.2–6.2)	38.2 (34.8–41.6)	24.9 (21.8–28.1)	32.1 (28.8–35.4)
50,000–99,999	1,380 (35.4)	5.4 (4.1–6.8)	39.1 (36.1–42.1)	21.1 (18.5–23.7)	34.3 (31.4–37.2)
≥100,000	926 (24.4)	3.7 (2.4–5.1)	46.8 (43.1–50.6)	20.6 (17.5–23.7)	28.8 (25.6–32.1)
**Marital status**					
Married or living with partner	2,586 (60.1)	5.0 (4.1–5.9)	39.8 (37.6–42.0)	22.1 (20.2–24.0)	33.1 (31.1–35.2)
Single	843 (26.5)	5.4 (3.6–7.2)	43.2 (39.3–47.1)	20.6 (17.4–23.9)	30.8 (27.3–34.4)
Divorced, widowed, or separated	698 (13.4)	6.0 (3.8–8.2)	34.0 (29.9–38.1)	21.1 (17.6–24.7)	38.9 (34.5–43.3)
**US Census region^f^**					
Northeast	725 (18.2)	5.3 (3.5–7.2)^d^	40.4 (36.3–44.6)	17.6 (14.3–20.8)	36.7 (32.6–40.7)
Midwest	1,051 (21.4)	4.8 (3.3–6.3)	44.0 (40.4–47.5)	18.8 (16.1–21.4)	32.5 (29.2–35.7)
South	1,450 (37.0)	6.2 (4.7–7.6)	35.7 (32.8–38.5)	22.9 (20.3–25.5)	35.3 (32.4–38.1)
West	901 (23.4)	4.2 (2.7–5.6)	42.5 (38.7–46.4)	25.0 (21.6–28.5)	28.3 (24.8–31.7)
**Children aged <18 y living in household^g^**					
Yes	1,310 (27.8)	5.7 (4.1–7.4)	41.5 (38.1–44.8)	22.9 (20.1–25.8)	29.9 (26.8–33.0)
No	2,804 (72.2)	5.0 (4.2–5.9)	39.4 (37.3–41.5)	21.0 (19.2–22.8)	34.5 (32.5–36.5)
**Cigarette smoking status** h					
Never smoker	2,285 (59.3)	2.6 (1.8–3.4)^d^	42.4 (40.0–44.8)	25.4 (23.3–27.5)	29.6 (27.4–31.8)
Current smoker	547 (13.7)	15.4 (11.9–18.9)	38.1 (33.2–43.0)	10.0 (6.8–13.2)	36.5 (31.7–41.2)
Former smoker	1,207 (26.9)	6.4 (4.8–8.0)	36.4 (33.3–39.5)	19.8 (17.2–22.5)	37.3 (34.2–40.5)
**EVP use^i^**					
Never user	3,518 (85.0)	3.0 (2.3–3.6)^d^	39.3 (37.4–41.2)	24.0 (22.3–25.7)	33.7 (31.9–35.5)
Current user	191 (4.8)	29.4 (21.9–36.9)	33.3 (25.2–41.4)	6.7 (2.7–10.7)	30.6 (22.4–38.8)
Former user	400 (10.2)	13.1 (9.3–16.9)	48.9 (43.2–54.6)	8.9 (5.4–12.3)	29.1 (24.1–34.0)

Abbreviation: CI, confidence interval.

a Data came from 2015 Styles, a series of seasonal national consumer surveys developed and fielded by Porter Novelli. Styles draws from KnowledgePanel, an Internet panel recruited by using address-based probability sampling to reach respondents regardless of landline telephone or Internet access (10). If needed, households are provided with a laptop computer and Internet access. Styles is conducted among a nationally representative sample of US adults aged 18 years or older.

b Defined as responding “no harm,” “little harm,” “some harm,” “a lot of harm,” or “don’t know” to the question “Do you believe that when children breathe the aerosol from other people’s electronic vapor products that it causes . . .?”

c Sum of sample observations may not add up to total population because of missing data.

d Significant χ^2^ test (*P* < .05) across harm-perception groups within the specified characteristic.

e Result not presented because relative standard error >30%.

f Northeast: Connecticut, Maine, Massachusetts, New Jersey, New Hampshire, New York, Pennsylvania, Rhode Island, and Vermont; Midwest: Illinois, Indiana, Iowa, Kansas, Michigan, Minnesota, Missouri, Nebraska, North Dakota, Ohio, South Dakota, and Wisconsin; South: Alabama, Arkansas, Delaware, District of Columbia, Florida, Georgia, Kentucky, Louisiana, Maryland, Mississippi, North Carolina, Oklahoma, South Carolina, Tennessee, Texas, Virginia, and West Virginia; West: Alaska, Arizona, California, Colorado, Hawaii, Idaho, Montana, Nevada, New Mexico, Oregon, Utah, Washington, and Wyoming.

g Defined as responding yes to having at least one child under the age of 18 years living in the household.

h Current cigarette smokers were defined as respondents who reported smoking at least 100 cigarettes during their lifetime and currently smoking cigarettes “every day” or “some days.” Former smokers were defined as respondents who reported having smoked at least 100 cigarettes in their lifetime and who reported smoking “not at all” at the time of survey. Never cigarette smokers were defined as respondents who reported not having smoked 100 cigarettes in their lifetime.

i Current EVP users were defined as respondents who reported using any of the following products in their lifetime and within the previous 30 days: “electronic cigarettes (e-cigarettes), such as Blu, 21st Century Smoke, or NJOY”; “electronic hookahs (e-hookahs), hookah pens, or vape pens, such as Starbuzz or Fantasia”; or “some other electronic vapor product such as electronic cigars (e-cigars) or electronic pipes (e-pipes).” Former EVP users were defined as respondents who reported using EVPs at least once in their lifetime and who reported not using EVPs in the previous 30 days. Never EVP users were defined as respondents who reported not using EVPs in their lifetime and not using EVPs in the previous 30 days.

Following adjustment, the odds of perceiving “no harm” from secondhand EVP aerosol exposure were greater among men than women (adjusted odds ratio [AOR], 2.6; 95% confidence interval [CI], 1.7–3.9) (Table 2). The odds of perceiving “no harm” were lower among non-Hispanic blacks (AOR, 0.4; 95% CI, 0.2–0.7), Hispanics (AOR, 0.4; 95% CI, 0.2–0.9), and non-Hispanic other race (AOR, 0.2; 95% CI, 0.1–0.8) than among non-Hispanic whites. The odds of perceiving “no harm” were greater among current (AOR, 4.1; 95% CI, 2.1–7.8) and former (AOR, 1.9; 95% CI, 1.2–2.8) cigarette smokers compared with never smokers. In addition, compared with never EVP users, the odds of perceiving “no harm” were greater among current (AOR, 17.9; 95% CI, 8.0–39.9), and former (AOR, 7.5; 95% CI, 3.9–14.2) EVP users.

**Table 2:** Factors Associated With Perception of Harm for Children Exposed to Second Aerosol From Electric Vapor Products (EVPs) Among US Adults Aged 18 Years or Older (Unweighted n = 4,127), Styles Survey,^a^ 2015

Characteristic	Perceived Harm^b^		
	No Harm, AOR (95% CI)	Little Harm or Some Harm, AOR (95% CI)	Don’t Know, AOR (95% CI)
**Sex**			
Female	1 [Reference]		
Male	**2.6 (1.7–3.9)**	**1.7 (1.4–2.1)**	**1.4 (1.1–1.7)**
**Age, y**			
18–24	1 [Reference]		
25–44	1.4 (0.7–3.0)	1.1 (0.7–1.5)	1.5 (1.0–2.2)
45–64	1.7 (0.8–3.4)	0.9 (0.6–1.3)	**1.6 (1.1–2.4)**
≥65	1.5 (0.7–3.4)	**0.6 (0.4–0.9)**	1.3 (0.8–1.9)
**Race/ethnicity**			
Non-Hispanic white	1 [Reference]		
Non-Hispanic black	**0.4 (0.2–0.7)**	0.7 (0.5–1.0)	**0.6 (0.4–0.9)**
Hispanic	**0.4 (0.2–0.9)**	**0.6 (0.4–0.8)**	**0.5 (0.3–0.7)**
Non-Hispanic other than white or black	**0.2 (0.1–0.8)**	**0.6 (0.4–0.8)**	**0.3 (0.2–0.5)**
**Education level**			
<High school graduate	1 [Reference]		
High school graduate	0.8 (0.4–1.5)	1.2 (0.8–1.7)	1.0 (0.7–1.6)
Some college	0.8 (0.4–1.8)	1.3 (0.9–2.0)	1.0 (0.6–1.5)
College degree or beyond	1.0 (0.5–2.0)	1.5 (1.0–2.2)	1.1 (0.7–1.6)
**Annual household income, $**			
<25,000	1 [Reference]		
25,000–49,999	0.6 (0.3–1.1)	1.0 (0.7–1.4)	0.8 (0.5–1.1)
50,000–99,999	0.9 (0.5–1.7)	1.2 (0.9–1.7)	1.0 (0.7–1.4)
≥100,000	0.8 (0.4–1.5)	1.4 (1.0–2.0)	0.9 (0.6–1.4)
**Marital status**			
Married or living with partner	1 [Reference]		
Single	1.4 (0.8–2.3)	1.0 (0.7–1.4)	1.1 (0.8–1.6)
Divorced, widowed, or separated	1.1 (0.6–1.8)	1.1 (0.8–1.5)	1.1 (0.8–1.6)
**US Census region^c^**			
Northeast	1 [Reference]		
Midwest	0.7 (0.4–1.2)	1.0 (0.7–1.4)	0.8 (0.6–1.1)
South	0.8 (0.5–1.4)	0.7 (0.5–1.0)	0.8 (0.6–1.0)
West	0.6 (0.3–1.2)	0.9 (0.6–1.2)	**0.6 (0.4–0.9)**
**Children aged <18 y living in household^d^**			
No	1 [Reference]		
Yes	1.2 (0.7–2.1)	0.8 (0.6–1.1)	0.8 (0.6–1.1)
**Cigarette smoking status^e^**			
Never smoker	1 [Reference]		
Current smoker	**4.1 (2.1–7.8)**	**1.7 (1.1–2.8)**	**2.2 (1.4–3.5)**
Former smoker	**1.9 (1.2–2.8)**	1.1 (0.8–1.4)	1.3 (1.0–1.7)
**EVP use^f^**			
Never	1 [Reference]		
Current	**17.9 (8.0–39.9)**	**2.3 (1.1–4.9)**	**2.3 (1.1–4.7)**
Former	**7.5 (3.9–14.2)**	**2.7 (1.7–4.5)**	**1.8 (1.1–3.0)**

Abbreviations: CI, confidence interval; AOR, adjusted odds ratio.

a Data came from 2015 Styles, a series of seasonal national consumer surveys developed and fielded by Porter Novelli. Styles draws from KnowledgePanel, an Internet panel recruited by using address-based probability sampling to reach respondents regardless of landline telephone or Internet access (10). If needed, households are provided with a laptop computer and Internet access. Styles is conducted among a nationally representative sample of US adults aged 18 years or older.

b Defined as responding “no harm,” “little harm,” “some harm,” “a lot of harm,” or “don’t know” to the question “Do you believe that when children breathe the aerosol from other people’s electronic vapor products that it causes . . .?” Those who responded “no harm,” “little harm” or “some harm,” or “don’t know” were each compared with those who responded “a lot of harm.” Multinomial logistic regression model was adjusted for all covariates listed in the table. Significant (*P* < .05) AORs are noted in bold.

c Northeast: Connecticut, Maine, Massachusetts, New Jersey, New Hampshire, New York, Pennsylvania, Rhode Island, and Vermont; Midwest: Illinois, Indiana, Iowa, Kansas, Michigan, Minnesota, Missouri, Nebraska, North Dakota, Ohio, South Dakota, and Wisconsin; South: Alabama, Arkansas, Delaware, District of Columbia, Florida, Georgia, Kentucky, Louisiana, Maryland, Mississippi, North Carolina, Oklahoma, South Carolina, Tennessee, Texas, Virginia, and West Virginia; West: Alaska, Arizona, California, Colorado, Hawaii, Idaho, Montana, Nevada, New Mexico, Oregon, Utah, Washington, and Wyoming.

d Defined as responding yes to having at least one child under the age of 18 years living in the household.

e Current cigarette smokers were defined as respondents who reported smoking at least 100 cigarettes during their lifetime and currently smoking cigarettes “every day” or “some days.” Former smokers were defined as respondents who reported having smoked at least 100 cigarettes in their lifetime and who reported smoking “not at all” at the time of survey. Never cigarette smokers were defined as respondents who reported not having smoked 100 cigarettes in their lifetime.

f Current EVP users were defined as respondents who reported using any of the following products in their lifetime and within the previous 30 days: “electronic cigarettes (e-cigarettes), such as Blu, 21st Century Smoke, or NJOY”; “electronic hookahs (e-hookahs), hookah pens, or vape pens, such as Starbuzz or Fantasia”; or “some other electronic vapor product such as electronic cigars (e-cigars) or electronic pipes (e-pipes).” Former EVP users were defined as respondents who reported using EVPs at least once in their lifetime and who reported not using EVPs in the previous 30 days. Never EVP users were defined as respondents who reported not using EVPs in their lifetime and not using EVPs in the previous 30 days.

The odds of perceiving “little harm” or “some harm” were greater among men than women (AOR, 1.7; 95% CI, 1.4–2.1), and lower among adults aged 65 years or older (AOR, 0.6; 95% CI, 0.4–0.9) compared with adults aged 18 to 24 years, as well as among Hispanics (AOR, 0.6; 95% CI, 0.4–0.8) and non-Hispanic other race (AOR, 0.6; 95% CI, 0.4–0.8) compared with non-Hispanic whites. The odds of perceiving “little harm” or “some harm” were greater among current smokers (AOR, 1.7; 95% CI, 1.1–2.8) compared with never smokers, and among current (AOR, 2.3; 95% CI, 1.1–4.9) and former (AOR, 2.7; 95% CI, 1.7–4.5) EVP users compared with never EVP users.

The odds of reporting “don’t know” were greater among men than women (AOR, 1.4; 95% CI, 1.1–1.7), and adults aged 45 to 64 years compared with adults aged 18 to 24 years (AOR, 1.6; 95% CI, 1.1–2.4). In contrast, the odds of reporting “don’t know” were lower among non-Hispanic blacks (AOR, 0.6; 95% CI, 0.4–0.9), Hispanics (AOR, 0.5; 95% CI, 0.3–0.7), and non-Hispanic races other than white or black (AOR, 0.3; 95% CI, 0.2–0.5) compared with non-Hispanic whites, as well as among adults living in the West (AOR, 0.6; 95% CI, 0.4–0.9) compared with those in the Northeast. The odds of reporting “don’t know” were greater among current smokers (AOR, 2.2; 95% CI, 1.4–3.5) than among never smokers and among current (AOR, 2.3; 95% CI, 1.1–4.7) and former (AOR, 1.8; 95% CI, 1.1–3.0) EVP users compared with never EVP users.

## Discussion

Our findings reveal that 22% of US adults believe that exposure to secondhand EVP aerosol causes a lot of harm to children, 40% believe it causes little or some harm, 5% believe it causes no harm, and an additional one-third of adults reported not knowing whether secondhand EVP aerosol exposure causes harm to children. Current cigarette smokers and EVP users had greater odds of reporting that exposure to secondhand EVP aerosol causes “no harm” or “little harm” or “some harm” to children compared with never cigarette smokers and never EVP users. However, scientific evidence indicates that EVP aerosol exhaled into the air potentially exposes nonusers to aerosolized nicotine and other harmful and potentially harmful substances, including heavy metals, ultrafine particulates, and volatile organic compounds (1–5). Nicotine exposure during periods of developmental vulnerability, from fetal through adolescent stages, may have multiple adverse health effects, including impaired brain and lung development (2–6). The fact that many adults did not know whether there are harms associated with children’s exposure to EVP aerosol, and some incorrectly believed there were no harms, underscores the importance of educating the public on the health risks of secondhand EVP aerosol exposure for nonusers, particularly children (4).

Variations in perceived harm were observed across sociodemographic groups, even after adjusting for current cigarette smoking and EVP use. More specifically, the odds of responding “no harm,” “little harm” or “some harm,” or “don’t know” was greatest among men and non-Hispanic whites. These variations in harm perceptions may be the result of multiple factors, including use of other forms of tobacco use among these individuals, marketing messages that downplay the potential health risks of EVP use or exposure to EVP aerosol, or factors related to social disapproval of smoking or EVP use (4,14). These sociodemographic differences suggest that public health education may be warranted to inform the public about the potential health risks of exposure to EVP aerosol, especially for children.

The 2016 Surgeon General’s Report on e-cigarette use among youth and young adults concluded that e-cigarette aerosol is not harmless and stated, “Smokefree indoor air policies should be updated to prohibit the use of both conventional cigarettes and e-cigarettes, thereby preserving standard for clean indoor air” (4). As of December 2016, eight states (California, Delaware, Hawaii, Maine, North Dakota, New Jersey, Oregon, and Utah) and 507 localities have enacted comprehensive smoke-free laws that prohibit conventional smoking and EVP use in all indoor areas of work sites, restaurants, and bars (15). However, most of the US population is not protected by laws that specifically prohibit EVP use (16). Momentum to protect nonusers, particularly children, from exposure to secondhand aerosol in public areas may be impeded by lack of knowledge about the potential harms of secondhand EVP aerosol as well EVP advertising messages. Some EVP marketing includes unproven claims of safety as well as statements that the products are exempt from smoke-free policies, which may not be true in all states (1). These messages could undermine clean indoor air standards by encouraging people to use EVPs as substitutes for conventional smoking in areas where conventional smoking is prohibited. Clean indoor air standards protect from exposure to secondhand smoke and aerosol and have been shown to help reduce initiation of smoking and use of tobacco among young people by denormalizing tobacco use. (17). In addition, because some e‑cigarettes (eg, cigalikes) are designed to mimic smoking, allowing EVP use in places where smoking is prohibited could complicate enforcement of smoke-free policies (15). In addition to public places, private settings such as homes and vehicles are locations where children may also be exposed to secondhand EVP aerosol. Across the United States, 18.9% of adults do not have voluntary smoke-free home rules and 26.4% do not have smoke-free vehicle rules (18). By establishing 100% smoke-free home and vehicle rules that also include EVPs, parents and caregivers can protect the health of their children and help prevent exposure to secondhand EVP aerosol among children in their care.

In addition to prohibiting EVP use in indoor areas, other population-based public health interventions are warranted to protect the public, particularly children, from possible harmful effects of secondhand EVP aerosol exposure. Strategies could include informing the public about the potential harmful effects of secondhand EVP aerosol (15). Promoting tobacco-free norms, fully enforcing smoke-free laws that include EVP use, and educating the public about the harms related to secondhand EVP exposure for children could reduce the risk of exposure in children (15). When addressing potential public health harms associated with EVP use and secondhand exposure to EVP aerosol, it is important to implement the strategies promoted by the US Surgeon General to prevent and reduce the use of all forms of tobacco, including tobacco price increases, comprehensive smoke-free laws that include EVPs, high-impact antitobacco media campaigns, barrier-free cessation treatment and services, and comprehensive statewide tobacco control programs (18). Using a comprehensive approach, as well as educating the public on the dangers of nicotine exposure among children and adolescents, could reduce secondhand EVP exposure among nonusers and the misperception that EVP aerosol is harmless.

The findings of this study are subject to some limitations. First, respondents were recruited from a list of web panelists, which may limit generalizability. However, Styles data were weighted to be nationally representative of the US adult population, and tobacco use estimates from Styles are consistent with other national household surveys of US adults (19). Second, because of the questions available on the Styles survey, former EVP use was ascertained using ever use as a threshold for regular use. Given that it was not possible to distinguish between former EVP users who routinely used the products and those who only briefly experimented with the products, this classification could introduce bias. Third, limitations related to questionnaire content and variations in question comparability prevented the assessment of harm perceptions by other tobacco product use, cigarette smoking behaviors (eg, quit attempts), and type of EVP use (eg, disposable, mid-sized, tank system). We attempted to study dual use; however, because the number of respondents that reported being current cigarette smokers and current EVP users in our sample was low (113 [2.9%] using weighted data), it was not possible to assess this measure in more nuance because of unstable estimates and large relative standard errors. Fourth, self-reporting of smoking status and EVP use could lead to misclassification bias; the validity of self-reported smoking is well-established (20), but the validity of self-reported EVP use is uncertain. Finally, because the EVP product landscape is rapidly changing, EVP use may be underestimated if EVP users referred to the products they used with terminology other than what was provided in the questionnaire. However, multiple EVP product examples and terminologies were included in the question to minimize the potential for misclassification bias.

In conclusion, current and former cigarette smokers and EVP users had greater odds than nonusers of perceiving no harm toward children exposed to EVP aerosol. Variations in harm perceptions were also observed across sexes and race/ethnicities. EVP use potentially exposes nonusers, including children, to aerosolized nicotine and other harmful substances (1–6). Therefore, clean air — free of both smoke and EVP aerosol — remains the standard to protect health. In coordination with a comprehensive approach to prevent and reduce secondhand smoke exposure and tobacco use by young people, efforts are warranted to educate the public, particularly current and former cigarette smokers and EVP users, about the potential health risks of secondhand EVP aerosol exposure among children.

## Post-Test Information

To obtain credit, you should first read the journal article. After reading the article, you should be able to answer the following, related, multiple-choice questions. To complete the questions (with a minimum 75% passing score) and earn continuing medical education (CME) credit, please go to http://www.medscape.org/journal/pcd. Credit cannot be obtained for tests completed on paper, although you may use the worksheet below to keep a record of your answers.

You must be a registered user on http://www.medscape.org. If you are not registered on http://www.medscape.org, please click on the “Register” link on the right hand side of the website.

Only one answer is correct for each question. Once you successfully answer all post-test questions, you will be able to view and/or print your certificate. For questions regarding this activity, contact the accredited provider, CME@medscape.net. For technical assistance, contact CME@medscape.net. American Medical Association’s Physician’s Recognition Award (AMA PRA) credits are accepted in the US as evidence of participation in CME activities. For further information on this award, please go to https://www.ama-assn.org. The AMA has determined that physicians not licensed in the US who participate in this CME activity are eligible for *AMA PRA Category 1 Credits™*. Through agreements that the AMA has made with agencies in some countries, AMA PRA credit may be acceptable as evidence of participation in CME activities. If you are not licensed in the US, please complete the questions online, print the AMA PRA CME credit certificate, and present it to your national medical association for review.

## Post-Test Questions

### Study Title: Perceptions of Harm to Children Exposed to Secondhand Aerosol From Electronic Vapor Products, Styles Survey, 2015

#### CME Questions

1. You are a seeing a 6-month-old female infant brought in by her parents. They report that they use e-cigarettes in the house several times per day after quitting combustible cigarettes approximately 18 months ago. When asked what they know about the risks associated with using e-cigarettes around their daughter, they dismiss any possible harms. Approximately what proportion of participants in the current research believed that secondary aerosols from electronic vapor products (EVPs) caused no harm or little harm?
  - 4%
  - 14%
  - 22%
  - 45%
2. In contrast, approximately what percentage of respondents in the current study believed that secondhand aerosols from EVPs could cause much harm?
  - 4%
  - 14%
  - 22%
  - 38%
3. Which of the following variables in the current study was most associated with the belief that secondary aerosols from EVPs cause little or no harm?
  - Male sex
  - Non-Hispanic black race
  - Hispanic ethnicity
  - Status as a never-smoker
4. What can you tell this couple about the use of e-cigarettes and the potential harms associated with secondary aerosols?
  - E-cigarettes contain only a liquid form of nicotine
  - Secondary aerosols do not contain nicotine
  - US public health officials have yet to comment on secondary aerosols from EVPs
  - Harm from secondary aerosols can come from inhalation, ingestion, or even absorption through the skin
